# The Effects of Bone Cement Volume in Percutaneous Vertebroplasty for Thoracolumbar Junction Vertebral Compression Fractures: A Clinical Comparative Study

**DOI:** 10.1155/2022/4230065

**Published:** 2022-07-22

**Authors:** Meng Wang, Bo Li, Yuren Wang, Shengdan Jiang, Gen Wen, Leisheng Jiang, Xinfeng Zheng

**Affiliations:** ^1^Department of Clinic of Spine Center, Xinhua Hospital, Shanghai Jiaotong University School of Medicine, Shanghai 200092, China; ^2^Department of Orthopaedic Surgery, Shanghai Jiao Tong University Affiliated Sixth People's Hospital, Shanghai 200233, China

## Abstract

We compared the outcomes of patients treated with different volumes of polymethyl methacrylate bone cement during percutaneous vertebroplasty (PVP) for thoracolumbar vertebral compression fractures. We performed a comparative, retrospective study of 316 patients who underwent PVP for a single-level thoracolumbar vertebral compression fracture. Patients were divided into two groups: group A (≤5 mL; *n* = 146) and group B (>5 mL; *n* = 170). The visual analogue scale (VAS) for pain and the Roland-Morris Disability Questionnaire (RDQ) scores were compared between the two groups at 1 week and at 1, 6, 12, and 24 months after PVP. The incidence of cement leakage into the intervertebral discs was evaluated by a postoperative lateral radiograph assessment. Patients were evaluated for new fractures 1 and 2 years after PVP or when new fractures were suspected. Among the 316 patients enrolled, 245 completed the clinical research. No difference between groups A and B in terms of the VAS, RDQ, and rate of complications at all time points after surgery was observed. The presence of intervertebral disc leakage was a relative risk (RR) for subsequent total vertebral fracture (RR, 6.42; 95% confidence interval (CI), 2.72-14.19; *P* < 0.0001) and adjacent vertebral fracture (RR, 8.03; 95% CI, 2.74-23.54; *P* = 0.0001). A high volume of bone cement may increase the rate of subsequent total and adjacent vertebral fractures. However, the occurrence of intervertebral disc leakage is the principal risk factor for these negative outcomes of PVP.

## 1. Introduction

Percutaneous vertebroplasty (PVP) is a minimally invasive technique that involves percutaneous injection of polymethyl methacrylate or calcium phosphate cement into the involved vertebral body under C-arm fluoroscopy. For patients with an osteoporotic vertebral compression fracture (VCF), PVP can provide rapid pain relief and restore vertebral height, as well as improve function and mobility, which decreases the risk of mortality and incidence of complications. The efficacy of PVP has been proven in a series of clinical studies and a few randomized, controlled trials [1–8]. However, there were studies indicating that when the patients with VCFs were under PVP, the probability of subsequent vertebral fractures was from 2.4% to 23% [9, 10] and 2/3 of them occurred in the adjacent vertebra [11, 12]. The volume of bone cement used is the most important factor to consider with regard to the therapeutic effect of PVP. *In vitro* biomechanical studies have not identified a benefit of a higher volume of cement in restoring vertebral stiffness and strength [13, 14] or the height of the vertebra [14]. In fact, asymmetric injection of a high volume of cement negatively transforms the biological characteristics of the vertebra [13]. In clinical reports, amounts of cement ranging from 1 to 12 mL have been used for PVP treatment of VCFs [15–17]. However, the appropriate volume of cement to be used remains unclear. For balloon kyphoplasty, Röder et al. [18] reported that a cement volume > 4.5 mL was more effective than ≤4.5 mL. Of note, Jin et al. [19] reported that a high volume of cement during PVP increased the risk of subsequent adjacent vertebral fractures, and a volume of 4.9 mL was the most appropriate to minimize this risk. Based on this information, we conducted a retrospective, comparative study to determine if PVP performed with a low volume of cement provided the same clinical outcomes as PVP performed with a high volume of cement for the treatment of osteoporotic VCFs.

## 2. Materials and Methods

Eligible patients were those who underwent PVP for osteoporotic VCFs in the Department of Orthopedic Surgery at Xinhua Hospital Affiliated with Shanghai Jiaotong University School of Medicine between January 2008 and December 2013. The inclusion criteria were as follows: age ≥ 50 years and a single, acute, or subacute, painful osteoporotic VCF with a clinical onset < 3 months or a chronic, unhealed, painful, osteoporotic VCF with a clinical onset ≥ 3 months confirmed by spinal radiography and by magnetic resonance or emission computed tomography imaging. The exclusion criteria were as follows: pathological fractures from myeloma, metastatic tumor, or infection; history of coagulation disorders; disruption of the posterior wall of the fractured vertebral body; presence of any neurological symptom; severe cardiopulmonary comorbidity; suspected underlying malignant disease; diseases affecting bone metabolism; and history of glucocorticoid or antiosteoporosis drugs. The level of the VCF was classified as thoracic (T6-T9), thoracolumbar (T10-L2), or lumbar (L3-L5). All the patients included in this study were required to undergo dual-energy X-ray absorptiometry examination to determine bone mineral density scores of the lumbar vertebrae (L1–4). Fractured vertebrae were excluded at *T*-score evaluation when the fracture was at L1-L4.

**Figure 1:**
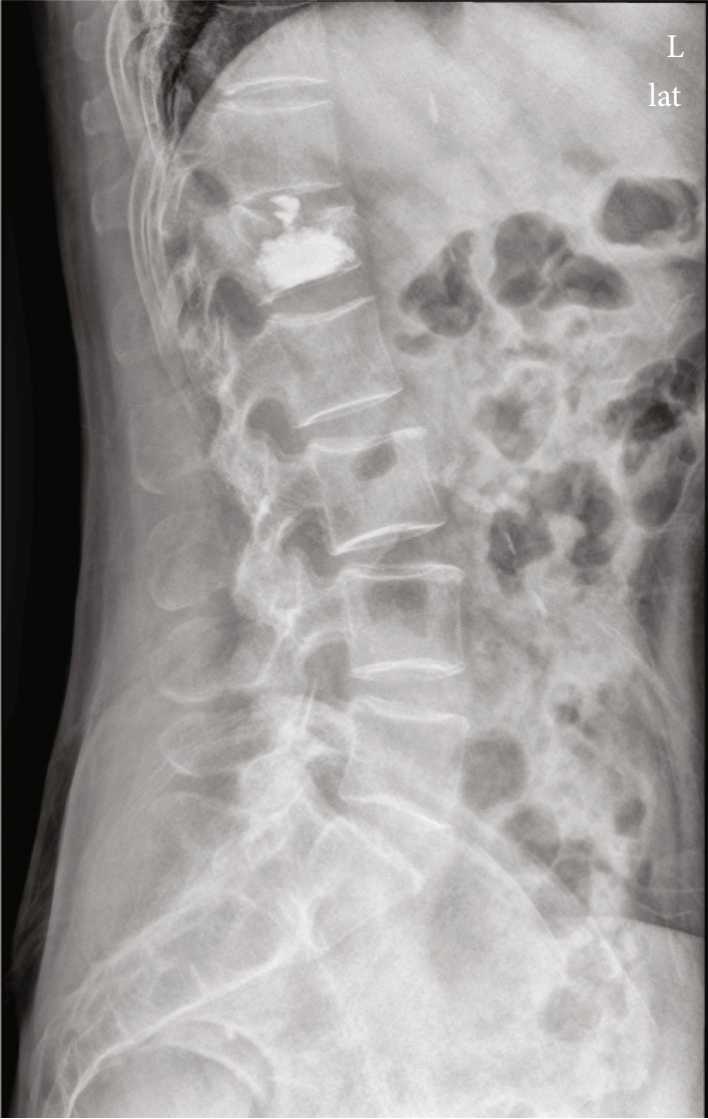
Cement leakage into the intervertebral disc on direct postoperative radiograph.

Patient follow-up was conducted at 1 week after PVP and at 1, 6, 12, and 24 months after PVP. Patient-reported scores on the visual analogue scale (VAS) for back pain and the Roland-Morris Disability Questionnaire (RDQ) for functional recovery were obtained at baseline before PVP and at each follow-up. A 10-point VAS was used to quantify pain intensity with anchors at “0” (no pain) and “10” (worst possible pain). The RDQ was used for scoring activities of daily life on a 23-point scale with physical disability worsening as the score increased. Cement leakage into the intervertebral disc was evaluated on a postoperative lateral radiograph (see Figure 1).

Evaluation for a new VCF was performed by magnetic resonance or radioisotope imaging at 1 and 2 years after PVP or when suspected from the clinical presentation.

A 5 mL volume of cement was used as the cut-off, which was based on the report by Jin et al. [19], to compare the low- and high-volume groups. Patients were classified into the appropriate group for analysis: a low-volume group (group A) received a volume of cement ≤ 5 mL, and a high-volume group (group B) received a volume of cement > 5 mL.

Continuous variables were reported as the mean and standard deviation (SD) or 95% confidence interval (CI), and categorical variables were reported as a number and percentage. For dichotomous variables, the risk ratio (RR) and 95% CI were calculated. The Student *t*-test was used to evaluate between-group differences for continuous variables, and the chi-squared (*χ*^2^) test was used for categorical variables. A *P* value < 0.05 was considered statistically significant.

The present study was approved by Xinhua Hospital, Shanghai Jiaotong University School of Medicine (approval number: XHEC-D-2020-071). Written informed consent was obtained from the patients.

## 3. Results

### 3.1. Baseline Characteristics

**Figure 2:**
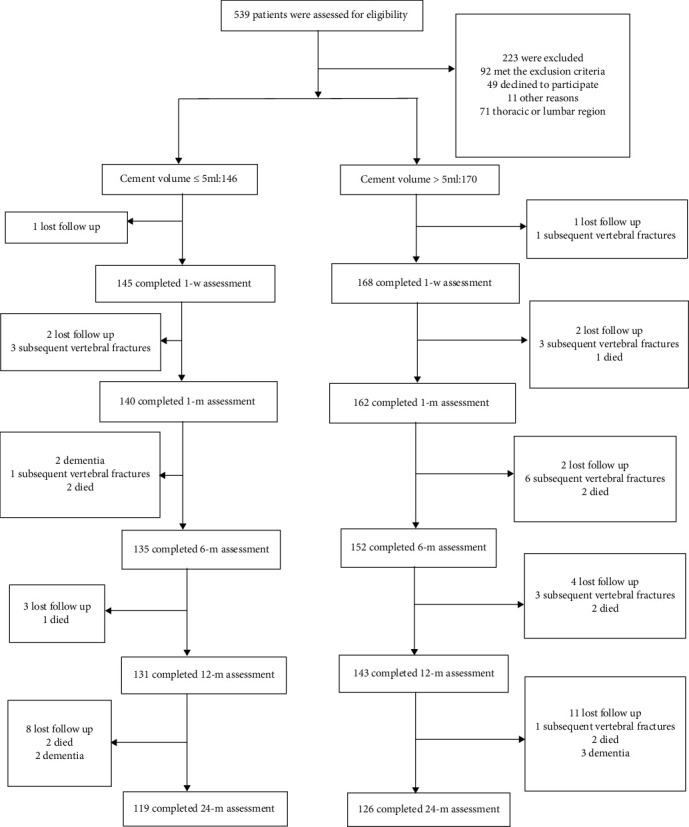
Flow chart of including, excluding, and dividing cases and 2-year follow-up.

A total of 539 patients from January 2008 to December 2013 were eligible for the study, and 387 of these patients met the inclusion criteria. The distribution of fractures was as follows: thoracic, *n* = 17; thoracolumbar, *n* = 316; and lumbar, *n* = 54. Owing to the small number of cases, thoracic and lumbar fractures were not included in our analysis to reduce the risk of bias (see Figure 2). The 316 patients with thoracolumbar fractures included 61 males and 255 females with an average age of 77.0 (range, 52 to 96) years; however, only 245 patients completed the 2-year follow-up from beginning to end (see Figure 2). The relevant characteristics of these patients, classified into groups A (≤5 mL) and B (>5 mL), are presented in Table 1.

### 3.2. VAS and RDQ

No difference between groups A and B with regard to the VAS and RDQ scores at each time point of measurement was observed (see Table 2).

### 3.3. Intervertebral Leakage and Subsequent Vertebral Fractures

A total of 35 (11.1%) patients had intervertebral disc leakage, 18 (5.7%) patients had subsequent total vertebral fracture, and 12 (3.8%) patients had subsequent adjacent vertebral fractures. The differences in these outcomes between groups A and B were as follows: intervertebral leakage, 13 (8.9%) and 22 (12.9%), respectively (*P* = 0.250); subsequent total vertebral fracture, 4 (2.7%) and 14 (8.2%), respectively (*P* = 0.036); and subsequent adjacent vertebral fracture, 2 (1.4%) and 10 (5.9%), respectively (*P* = 0.029) (see Table 3).

### 3.4. A Risk Factor to Subsequent Vertebral Fractures

Intervertebral disc leakage (*n* = 35) increased the risk of subsequent fractures. The difference between those patients with and without leakage was as follows: subsequent total vertebral fracture, 8 (22.9%) and 10 (3.6%), respectively (RR, 6.42; 95% CI, 2.72 to 15.19; *P* < 0.0001), and subsequent adjacent vertebral fracture, 6 (17.1%) and 6 (2.1%), respectively (RR, 8.03; 95% CI, 2.74 to 23.54; *P* = 0.0001) (see Table 4).

## 4. Discussion

A consensus regarding the effects of bone cement volume on the clinical efficacy of PVP for thoracolumbar VCFs does not exist. In our study, both low and high volumes of bone cement effectively relieved pain and promoted early recovery of function. Although a high volume of cement was associated with a high rate of subsequent total and adjacent vertebral fractures, the risk for these fractures was actually associated with the incidence of intervertebral leakage after surgery.

The effects of bone cement on the biomechanics of vertebrae have previously been reported. Belkoff et al. [20] reported that 2 mL of cement increased the strength of fractured vertebrae at the thoracic, lumbar, and thoracolumbar level; however, improvement in the rigidity of the vertebrae was specific to the level: 4 mL was required at the thoracic level, 4-6 mL was required at the lumbar level, and 4-8 mL was required at the thoracolumbar level, with the volume dependent on the cement materials used. Molloy et al. [14] reported that, although 3.5 mL of bone cement was sufficient to strengthen a vertebral fracture, a 7.0 mL volume was more effective; again, both volumes of bone cement failed to effectively repair the rigidity of vertebrae. Moreover, the effects of bone cement on osteoporotic bone tissue and, ultimately, on fracture repair have been an issue of long-standing controversy. In an animal study, Hu et al. [21] demonstrated that bone cement might not cause permanent injury to the bone tissue but could prolong the repair cycle of the bone surface.

The mechanism by which PVP yields its analgesic effects remains uncertain, although several hypotheses have been proposed and are worth exploring [22–24]. The first is that bone cement immediately solidifies the vertebra after injection and provides fixation to the fracture, which eliminates the fracture-site micromovements that stimulate sensory nerve endings. The second is that the heat effect of bone cement and cytotoxicity of monomers damage the sensory nerve endings, thus decreasing pain. The third is that the pain relief could be mediated by the local anesthetics used during PVP. The fourth is the possibility that PVP provides a placebo effect. Obviously, the last three hypotheses do not have an association with the volume of bone cement used. As such, the first hypothesis would clarify the association between pain improvement and postoperative vertebral strength. *In vitro* studies have proven that a low volume of bone cement is not inferior to a high volume for restoring the strength of a vertebra [13, 14, 20], which would explain why the volume of cement is not associated with the pain relief effect of PVP. In the same way, recovery of daily function depends on both the improvement of pain and restoration of vertebral strength and, thus, the volume of cement would have little effect on recovery of function. In contrast, the findings of Nieuwenhuijse et al. [22] showed that the degree of pain relief after PVP was related to the volume of bone cement, and a volume equivalent to 24% of the volume of the vertebral body provided the optimal effect. In our experience, alleviation of pain can be achieved effectively using both a low and high volume of cement. Luo et al. [23] reported that a 3.5 mL volume of cement maximizes recovery of normal stress distribution across both the fractured and adjacent vertebrae.

Several studies have confirmed that, compared to conservative treatment, vertebroplasty does not increase the incidence of subsequent fractures [24–29], but other studies have concluded the opposite [1, 30]. We note that a low volume of cement was used in two of the studies that reported the risk of subsequent fractures from PVP [25, 29]. In addition, we note that, in another study that reported an increased risk of subsequent fractures from PVP, the volume of cement was not specifically stated but the description provided was a fractured vertebra “fully filled” with bone cement [30]. The systematic review by Han et al. [31] reported that the volume of bone cement had no effect on the incidence of subsequent fractures. However, the studies included in Han et al.'s systematic review had wide variations in the volume of cement used, which resulted in high heterogeneity and an inability to conclusively determine the differential effects of a low or high volume. We further note that there is little evidence regarding the effect of different volumes on thoracolumbar VCFs after PVP. The volume of the vertebral body varies greatly across different segments of the spine; therefore, the possible differential effects of the volume of cement used must be examined at each level of the spine. We know that certain factors influence the incidence of subsequent fractures after PVP, namely, bone mineral density, body mass index, and bone cement intervertebral disc leakage [32, 33]. Our study adds to these findings by providing evidence that intervertebral disc leakage after PVP is a significant risk factor for subsequent vertebral fractures.

Our study shows that the incidence of intervertebral disc leakage after PVP is higher when a high volume of bone cement is used than when a low volume of bone cement is used, but the difference was not significant. Our study did confirm, however, that intervertebral disc leakage of bone cement is a risk factor for subsequent fractures.

Liu et al. [34] proposed that subsequent vertebral fractures after PVP reflected the natural progression of osteoporosis; this could explain the association between higher volumes of bone cement and an increased risk of subsequent fracture of adjacent but not distal vertebrae [35]. Syed et al. [36] confirmed that the use of a low volume of cement did not correlate with the distribution of subsequent vertebral fractures or incidence of intervertebral disc leakage. Of clinical importance, the volume of bone cement used plays an important role in improving the kyphotic deformity that results from VCFs of the thoracic spine.

The effects of bone cement on the stress of intervertebral discs have also been a controversial issue. Although bone cement intervertebral disc leakage is an important factor for subsequent fractures, the *in vitro* study by Aquarius et al. [37] indicated that bone cement does not increase peak stress of the lamina terminalis and, thus, would not cause an adverse change in the stress on adjacent vertebrae. However, Zhao et al. [38] indicated that bone cement could induce intervertebral disc degeneration, and both a higher volume of bone cement and longer time lapse since PCP resulted in more severe intervertebral disc degeneration; moreover, their research indicated more severe disc degeneration with the use of polymethyl methacrylate than with calcium phosphate cement.

Our study has several limitations. Foremost is the retrospective design of the study, which prevents an inference of any causality. Second is the limited number of cases of thoracic and lumbar VCFs, which prevented us from including these spinal levels in our analysis. Therefore, our findings are only applicable for thoracolumbar VCFs. Future research is warranted to examine the differential outcomes for thoracic and lumbar VCFs. Regarding our study design, we used a pairwise observation instead of a factor analysis and, thus, the specific effects of the volume of bone cement on measured outcomes require further analysis. Large, multicenter, randomized, controlled trials are needed to provide the necessary evidence regarding the optimal volume of bone cement on the efficacy of PVP for VCFs.

No clear difference between the use of low and high volumes of bone cement on the clinical outcomes of PVP for thoracolumbar VCFs was observed in our study. The volume of bone cement used did not influence the incidence of cement leakage into the intervertebral disc. However, intervertebral disc leakage was identified as a specific risk factor for adjacent vertebral fractures.

## 5. Conclusions

Both low and high volumes of bone cement effectively relieved pain and promoted early recovery of function in our study. A high volume of bone cement may increase the rate of subsequent total and adjacent vertebral fractures. However, the occurrence of intervertebral disc leakage is the principal risk factor for these negative outcomes of PVP.

## Figures and Tables

**Table 1:** Baseline characteristics of the 316 patients with thoracolumbar OVCFs treated with different volumes of cement.

	Low dose (≤5 mL)	High dose (>5 mL)	*P* value
Number of patients	146	170	
Sex, female; *N* (%)	116 (79.5%)	139 (81.8%)	0.606
Age, years; mean (SD)	77.1 (8.4)	76.2 (8.5)	0.305
BMD (*T*-value); mean (SD)	-2.7 (0.6)	-2.8 (0.7)	0.062
BMI (kg/m^2^); mean (SD)	22.5 (2.8)	23.1 (3.7)	0.112
Cement volume, mL; mean (SD)	4.4 (0.8)	6.0 (0.6)	<0.01
Cement volume; *N*			
2.1-4.0 mL	43		
4.1-5.0 mL	103		
5.1-6.0 mL	151		
6.1-8.0 mL	14		
8.1-10.0 mL	5		

BMD: bone mineral density; BMI: body mass index; OVCFs: osteoporotic vertebral compression fractures.

**Table 2:** The VAS and RDQ scores at the different time points of measurement.

	Low-dose (≤5 mL)		High-dose (>5 mL)		*P* value
	*N*	Mean (SD)	*N*	Mean (SD)	
VAS at different periods					
Initial	146	7.7 ± 1.1	170	7.6 ± 1.0	0.489
1 w	145	4.8 ± 1.6	168	4.8 ± 1.8	0.686
1 m	140	4.2 ± 1.9	162	3.9 ± 1.7	0.179
6 m	135	3.8 ± 2.0	152	3.6 ± 1.7	0.255
12 m	131	3.5 ± 1.7	143	3.8 ± 1.8	0.182
24 m	119	3.6 ± 1.5	126	3.5 ± 1.3	0.291
RDQ at different periods					
Initial	146	19.0 ± 1.8	170	18.9 ± 2.0	0.689
1 w	145	14.1 ± 2.8	168	13.7 ± 3.2	0.249
1 m	140	12.6 ± 4.1	162	12.0 ± 3.8	0.193
6 m	135	11.8 ± 3.4	152	11.5 ± 3.4	0.383
12 m	131	11.5 ± 4.0	143	11.1 ± 4.0	0.417
24 m	119	11.4 ± 4.0	126	11.6 ± 4.1	0.570

VAS: visual analogue scale; RDQ: Roland-Morris Disability Questionnaire.

**Table 3:** The IDL, STVF, and SAVF in respective groups with different volumes of cement.

	≤5 mL	>5 mL	RR	*P* value
IDL	13/146	22/170	1.45	0.250
STVF	4/146	14/170	3.01	0.036
SAVF	2/146	10/170	4.29	0.029

IDL: intervertebral disc leakage; STVF: subsequent total vertebral fracture; SAVF: subsequent adjacent vertebral fracture; RR: relative risk.

**Table 4:** The independent risk factor analysis of intervertebral disc leakage for subsequent vertebral fracture.

	With IDL	Without IDL	RR	*P* value
STVF	8/35	10/281	6.42	*P* < 0.0001
SAVF	6/35	6/281	8.03	*P* = 0.0001

IDL: intervertebral disc leakage; STVF: subsequent total vertebral fracture; SAVF: subsequent adjacent vertebral fracture; RR: relative risk.

## Data Availability

The data used to support the findings of this study are included within the article.
